# Supplementary material for: Analysing how changes in the health status of healthcare workers affects epidemic outcomes
Source: Epidemiol Infect. 2021 Feb 8;149:e42. doi: 10.1017/S0950268821000297 (PMC7925990; doi:10.1017/S0950268821000297)
Supplement: Supplementary file 1 [file S0950268821000297sup001.docx]

***Analyzing How Changes in the Health Status of Healthcare Workers Affects Epidemic Outcomes***

***SUPPLEMENTARY MATERIAL***

**A. Alternate models: overview and model equations**

The full model (main text Fig. 1A and Eqs. 1-9) extends an SIR-type^13^ model with a vaccinated class, healthcare workers (HCWs) as a distinct population, and accounting for the quality of care, $Q(P(t))$, provided by the uninfected fraction of HCWs, $P(t)$. Since our aim is to highlight the impact that quality of care delivered in the course of an epidemic may have on outcomes, we contrast this full model with alternate models that relax those assumptions, specifically whether we should account for quality of care (alternate models II and III neglect quality of care), or account for HCWs as a distinct population (alternate models I and III model HCWs as a fixed initial proportion of the total population). In the main text, we outlined and discussed the full model and how we model quality of care as a function of healthcare worker population, providing only brief descriptions of alternate models. Here we provide details and differential equations describing alternate models I-III. Table S1 shows how the four models are related.

With best quality of care (*Q* = 1, or quality of care at 100%), infected individuals, *I*, may die before recovering with probability *ρ* or recover with probability 1-*ρ*, at recovery rate *γ*. The probability of dying before recovery has both upper and lower bounds dependent on *ρ* when dynamic quality of care is considered. When quality of care is 100%, the probability of dying before recovery is *ρ*, and when it is 0%, the probability of dying before recovery is 1/(2-*ρ*). Similarly, the recovery rate varies with dynamic quality of care, from *γ* when quality of care is 100%, to 0 when quality of care is 0%. These parameters, describing probability of death and recovery rate, would remain constant if you neglect the effects of quality of care. We assume that once infected individuals have recovered, *R*, they are no longer susceptible, and will not lose immunity in the time frame modelled. Further, we assume that vaccines grant lasting immunity to the disease as well, so the susceptible individuals, vaccinated at rate *ν*, will remain in the vaccinated class, at least for the duration of the outbreak under consideration as vaccinated individuals, *V*.

**A1. Alternate model I**

In the full model we made a distinction between the general population and HCWs, since HCWs are often subject to different dynamics, for example more frequent exposure to infected individuals or higher vaccination rates. To illustrate the importance of this distinction, in alternate model I (Fig. 1B) we eliminate this assumption, and consider a single homogeneous population. Differential equations describing alternate model I (Fig. 1B) are:

|  | $\dot{S}\left( t \right)=-\frac{\beta\left( I\left( t \right) \right)}{N\left( t \right)}S(t)- Q(P(t))rvS\left( t \right)$ | (Eq S1) |
| --- | --- | --- |
|  | $\dot{I}\left( t \right)=\frac{\beta\left( I\left( t \right) \right)}{N\left( t \right)}S(t)- \frac{\gamma I\left( t \right)Q\left( P\left( t \right) \right)}{\left( 1-\rho\right)Q\left( P\left( t \right) \right)}$ | (Eq S2) |
|  | $\dot{R}\left( t \right)=Q\left( P\left( t \right) \right)\gamma I\left( t \right)$ | (Eq S3) |
|  | $\dot{V}\left( t \right)=Q\left( P\left( t \right) \right)rvS\left( t \right)$ | (Eq S4) |
|  | $N\left( t \right)=S\left( t \right)+I\left( t \right)+R\left( t \right)+V\left( t \right)$ | (Eq S5) |

As described in the main text, quality of care $Q(P(t))$ depends on the uninfected fraction of the HCW population which are directly accounted for in alternate model I. We therefore assume instead that the HCW uninfected fraction is equivalent to the total uninfected fraction, $P(t)=\left( \frac{S(t)+R(t)+V(t)}{S(0)+I(0)+R(0)+V(0)} \right)$. It is through the quality of care that we assume modeling HCWs as a distinct population will affect epidemic outcomes. Recall that the quality of care $Q(P(t))$ modulates the rate at which the susceptible population is vaccinated, *v,* probability of dying, $\rho,$ as well as the rate at which individuals recover, $\gamma$.

**A2. Alternate model II**

In alternate model II (Fig. 1C), as in the full model, we aim to capture distinct dynamics between the general population and HCWs by modeling them separately. However, we neglect the potential decline in of quality of care delivered as the epidemic affects the HCW population. The system of differential equations for alternate model II is therefore

|  | $\dot{S}\left( t \right)=-\frac{\beta\left( I\left( t \right)+I_{h}\left( t \right) \right)}{N\left( t \right)}S(t)- rvS\left( t \right)$ | (Eq S6) |
| --- | --- | --- |
|  | $\dot{I}\left( t \right)=\frac{\beta\left( I\left( t \right)+I_{h}\left( t \right) \right)}{N\left( t \right)}S\left( t \right)- \frac{\gamma I\left( t \right)}{\left( 1-\rho\right)}$ | (Eq S7) |
|  | $\dot{R}(t)=\gamma I\left( t \right)$ | (Eq S8) |
|  | $\dot{V}\left( t \right)=rvS\left( t \right)$ | (Eq S9) |
|  | $\dot{S}_{h}\left( t \right)=-\frac{\beta_{h}\left( I\left( t \right)+I_{h}\left( t \right) \right)}{N\left( t \right)}S_{h}(t)- vS_{h}\left( t \right)$ | (Eq S10) |
|  | $\dot{I}_{h}(t)=\frac{\beta_{h}\left( I\left( t \right)+I_{h}\left( t \right) \right)}{N\left( t \right)}S_{h}(t)- \frac{\gamma I_{h}\left( t \right)}{\left( 1-\rho\right)}$ | (Eq S11) |
|  | $\dot{R}_{h}\left( t \right)=\gamma I_{h}\left( t \right)$ | (Eq S12) |
|  | $\dot{V}_{h}\left( t \right)=vS_{h}\left( t \right)$ | (Eq S13) |
|  | $N\left( t \right)=S\left( t \right)+I\left( t \right)+R\left( t \right)+V\left( t \right)+S_{h}\left( t \right)+I_{h}\left( t \right)+R_{h}\left( t \right)+V_{h}\left( t \right)$ | (Eq S14) |

Note that for the purposes of our illustration, HCWs receive vaccination at twice the rate of the general population. In general, this may not be the case.

**A3. Alternate model III**

Alternate model III is the simplest baseline model, an SIR-type model with an additional vaccinated class, neglecting the impact of HCWs or quality of care, shown schematically in Fig. 1D. This model is described by the following system of differential equations:

|  | $\dot{S}\left( t \right)=-\frac{\beta\left( I\left( t \right) \right)}{N\left( t \right)}S(t)- rvS\left( t \right)$ | (Eq S15) |
| --- | --- | --- |
|  | $\dot{I}\left( t \right)=\frac{\beta\left( I\left( t \right) \right)}{N\left( t \right)}S(t)- \frac{\gamma I\left( t \right)}{\left( 1-\rho\right)}$ | (Eq S16) |
|  | $\dot{R}\left( t \right)=\gamma I\left( t \right)$ | (Eq S17) |
|  | $\dot{V}\left( t \right)=rvS\left( t \right)$ | (Eq S18) |
|  | $N\left( t \right)=S\left( t \right)+I\left( t \right)+R\left( t \right)+V\left( t \right)$ | (Eq S19) |

While we presented the alternate models I-III in order of decreasing complexity from the full model, alternate models I-II (Eqs. S1-S5, S6-S14, respectively) and the full model (main text, Eqs. 1-9) represent extensions of alternate model III.

|  | Health Care Workers explicit | Health Care Workers in general population |
| --- | --- | --- |
| Quality of Care | Full model (Fig. 1 A) | Alternate Model I (Fig. 1B) |
| No Quality of Care | Alternate Model II (Fig. 1C) | Alternate Model III (Fig. 1D) |

Table S1. The four related models considered in this study. See also Fig. 1.

**B. Further discussion of the Quality of Care function,** $\boldsymbol{Q(P(t))}$

Our modeling aims to investigate the quality of care delivered by healthcare workers (HCWs), how it may change as HCWs themselves become infected, and whether this has consequences for predictions of infection case counts and deaths. There are no empirical measurements for quality of care. We therefore chose to employ a sigmoid function, as this function can incorporate both linear and nonlinear changes to the quality of care as the proportion of uninfected individuals or uninfected healthcare workers change. We described this function in the main text (see **2.3**). Here we discuss the different parameterizations of the model, as well as the impact of the normalization that we impose.

**B1. Quality of care parameterization**

We model the quality of care, $Q\left( P\left( t \right) \right)$, as a function of the proportion of HCWs, $P\left( t \right)$, that are uninfected and able to provide care. We choose $Q\left( P\left( t \right) \right)$ to be a sigmoidal function, $1/\left( 1+e^{-k\left( P\left( t \right)-(1-m) \right)} \right),$ that is normalized such that $Q\left( 1 \right)= 1$, and $Q\left( 0 \right)= 0$, and interpret the two shape parameters associated with $Q\left( P\left( t \right) \right)$, $k$ and $m$*,* as describing loss impact ($k$) and the health system redundancy ($m$). This allows us to showcase healthcare systems working at levels from 0% to 100%.

|  | $Q\left( t \right)=Q\left( P\left( t \right) \right)=\frac{\frac{1}{1+e^{-k\left( P\left( t \right)-(1-m) \right)}}-\frac{1}{1+e^{k(1-m)}}}{\frac{1}{1+e^{-k\left( m \right)}}-\frac{1}{1+e^{k(1-m)}}}.$ | (Eq S20) |
| --- | --- | --- |


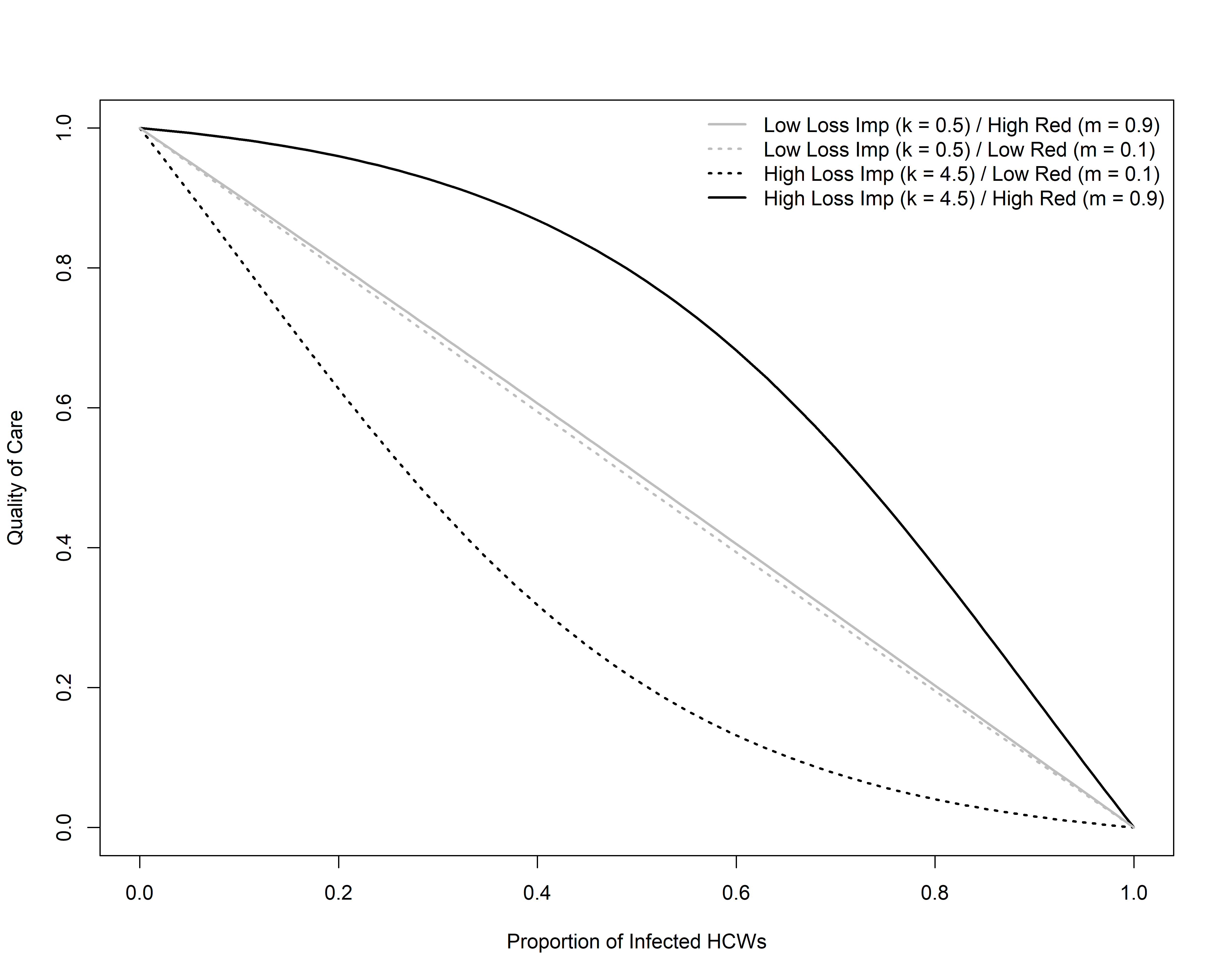


*Figure S1: Sample shapes of the quality of care function* $Q(P(t))$ *as a function of the proportion of infected HCWs, 1-P(t). The quality of care function depends on the value of the loss impact parameter,* $k$*, and the redundancy parameter,* $m$*. Each curve illustrates a sample quality of care function belonging to one of the four quadrants in Fig. 3. We take representative values k=0.5 and k=4.5 to model low and high loss impact, respectively, and m=0.1 and m=0.9 to model low and high redundancy, respectively.*

Collectively, the two shape parameters, $k$ and $m$, can represent a robust healthcare system (high loss impact (high $k$), high redundancy (high $m$)), a fragile system (high $k$, low $m$) and other combinations in between, as illustrated in Fig. S1.

We note that the normalization of the quality of care function to bound the values between 0 and 1 leads to a somewhat counter-intuitive response to the loss impact parameter, $k$. While we would expect a higher loss impact parameter $k$ to be strictly associated with a weaker health system, *k* and *m* interact, and the redundancy parameter $m$ dominates for high loss impact: the normalization (see Eq. S20) means that even when $k$ is high, there is little change in quality of care until a threshold in redundancy $m$ is reached. We explore this further in the next section and Fig. S2. Through these two shape parameters, the quality of care function $Q(t)$ can describe multiple modes of decline. This flexibility is important, as the function itself has no empirical estimate, and no supporting observations. As shown in Figure 3 in the main text, we consider loss impact parameter values ($k$) between 0-5 and redundancy parameter values ($m$) between 0 and 1, and we test the sensitivity of epidemic outcomes as a function of $k$ and $m$.

The quality of care function shows a range of behaviors as it spans a concave to convex region which can be used to capture healthcare systems of varying strengths (Fig. S1). High loss impact (high $k$) and low redundancy (low $m$) result in a quality of care that declines steeply after just a few healthcare workers are infected (solid black line Fig. S1), while high loss impact (high $k$) and high redundancy (high $m$) mean that quality of care declines gradually until many healthcare workers are infected, and only then declines steeply (dotted black line in Fig. S1). Low loss impact yields a quality of care delivered that declines in an intermediate manner regardless of the redundancy parameter (solid and dotted grey lines in Fig. S1).

**B2. Impact of quality of care normalization**

In order to limit the range of quality of care $Q(P(t))$ as a function ranging from 0 to 1, with $Q(P(t))=1$ indicating a health system at full strength, and $Q(P(t))=0$ as a fully depleted health system, we imposed a normalization (Eq. S20 and main text Eq. 10). The normalization leads to counterintuitive behavior given high loss impact (high $k$) in the quality of care function. Intuitively, one may assume that a higher loss impact per worker would lead to rapidly decaying quality of care function, but since the redundancy parameter dominates outcomes, this is not what is observed: rapid drop off only occurs after enough personnel have been lost. Examples to better understand the relationship between the two shape parameters $k$ and $m$, taking one fixed while varying the other, are provided in Fig. S2.


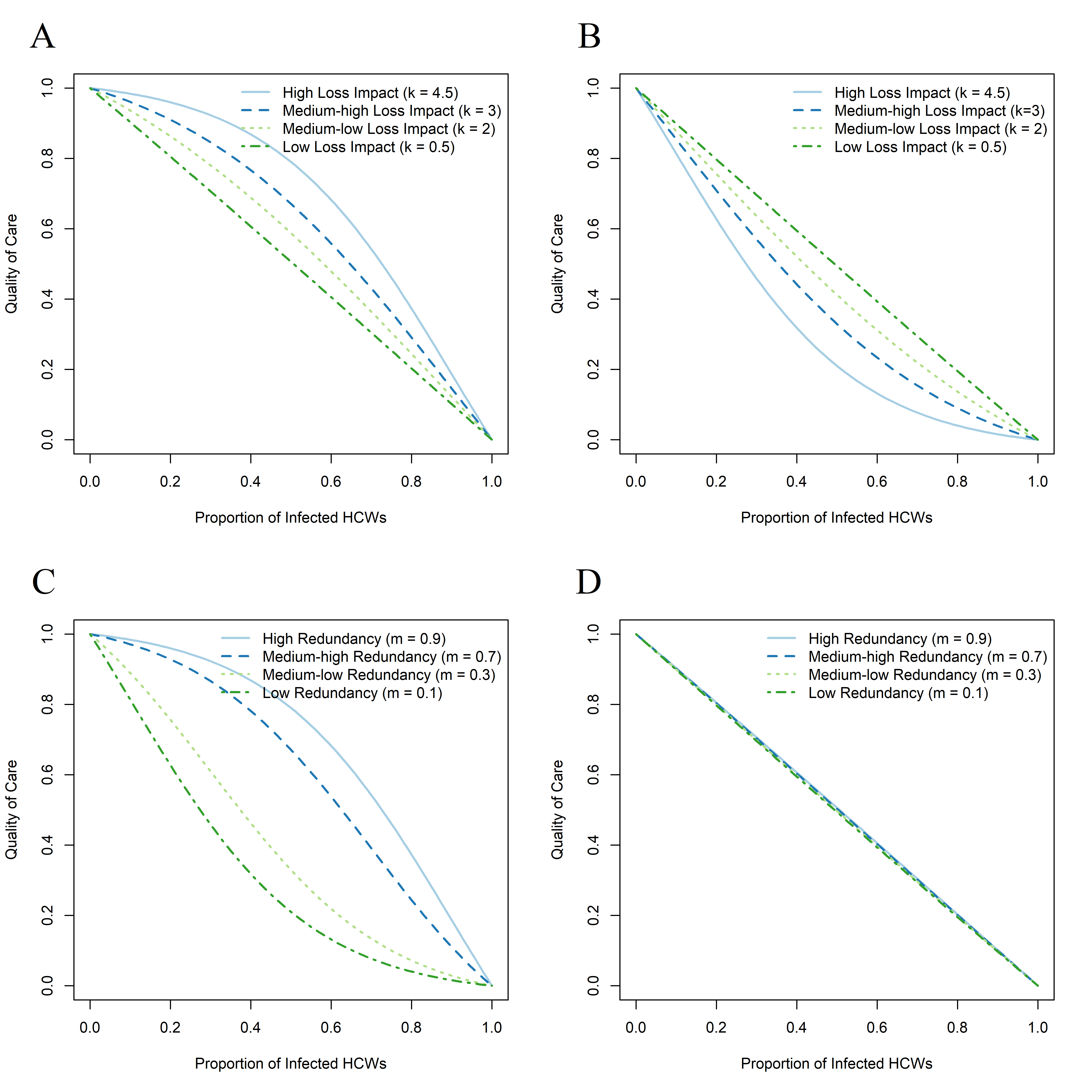


*Figure S2: Quality of care functions Q(P(t)) (Eq. S20), as a function of the proportion of infected HCWs 1-P(t), depending on the loss impact parameter, k, and redundancy parameter, m.*

A) *Quality of care assuming high redundancy (m=0.9), while the loss impact is varied from low to high levels (k=0.5, 2, 3, 4.5).*

B) *Quality of care assuming low redundancy (m=0.1), while the loss impact is varied from low to high levels (k=0.5, 2, 3, 4.5).*

C) *Quality of care assuming high loss impact (k=4.5), while redundancy is varied from high to low levels (m=0.9, 0.7, 0.3, 0.1).*

D) *Quality of care assuming low loss impact (k=0.5), while redundancy is varied from high to low levels (m=0.9, 0.7, 0.3, 0.1).*

For fixed, high redundancy (modeled with high $m$), and varying the loss impact parameter, we observe that the higher loss impact leads to the most favorable quality of care, i.e. highest as the proportion of infected HCWs increases, relative to lower loss impact (Fig. S2A). If instead we fix model redundancy to low levels (modeled with low $m$), we see that a higher loss impact (high $k$) yields the worst quality of care, while the lower loss impact (low $k$) now yields the best quality of care delivered as the infected HCW fraction increases (Fig. S2B). It should be noted that all of the quality of care functions in case of low redundancy (Fig. S2B) are all worse than in the case of high redundancy (Fig. S2A).

When we have a fixed high loss impact, modeled with high $k$, and varying the redundancy parameter, modeled by varying $m$, we observe that the quality of care provided decreases as the redundancy worsens ($m$ decreases). That is, high redundancy leads to the best quality of care for any HCW proportion compared to low redundancy, which leads to the worst quality of care for any HCW proportion (Fig. S2C). Additionally, when we have fixed low loss

impact, modeled with low $k$, and varying the redundancy parameter, again the best resultant quality of care function for any HCW proportion is induced by the higher redundancy while the worst is caused by the lower redundancy (Fig. S2D). It is important to note that the quality of care for the two highest levels of redundancy (modeled with $m=0.9$ and 0.7) was higher when the loss impact was fixed to a high value, but for the lower levels of redundancy (modeled with ($m=0.3$ and 0.1), higher quality of care was associated with loss impact fixed to a lower values.

In conclusion, normalization of the sigmoidal function to generate a quality of care function $QP(t))$ (Eq. S20) seems to contradict intuition on the effect of loss impact parameter on decline in the quality of care function. This has been showcased in the breakdown of the 4 scenarios shown in Fig. S2. Under the normalization, when simultaneously varying parameters that model loss impact and redundancy, the redundancy dominates changes in the quality of care function for smaller fractions of infected HCWs.

**C. Sensitivity Analyses**
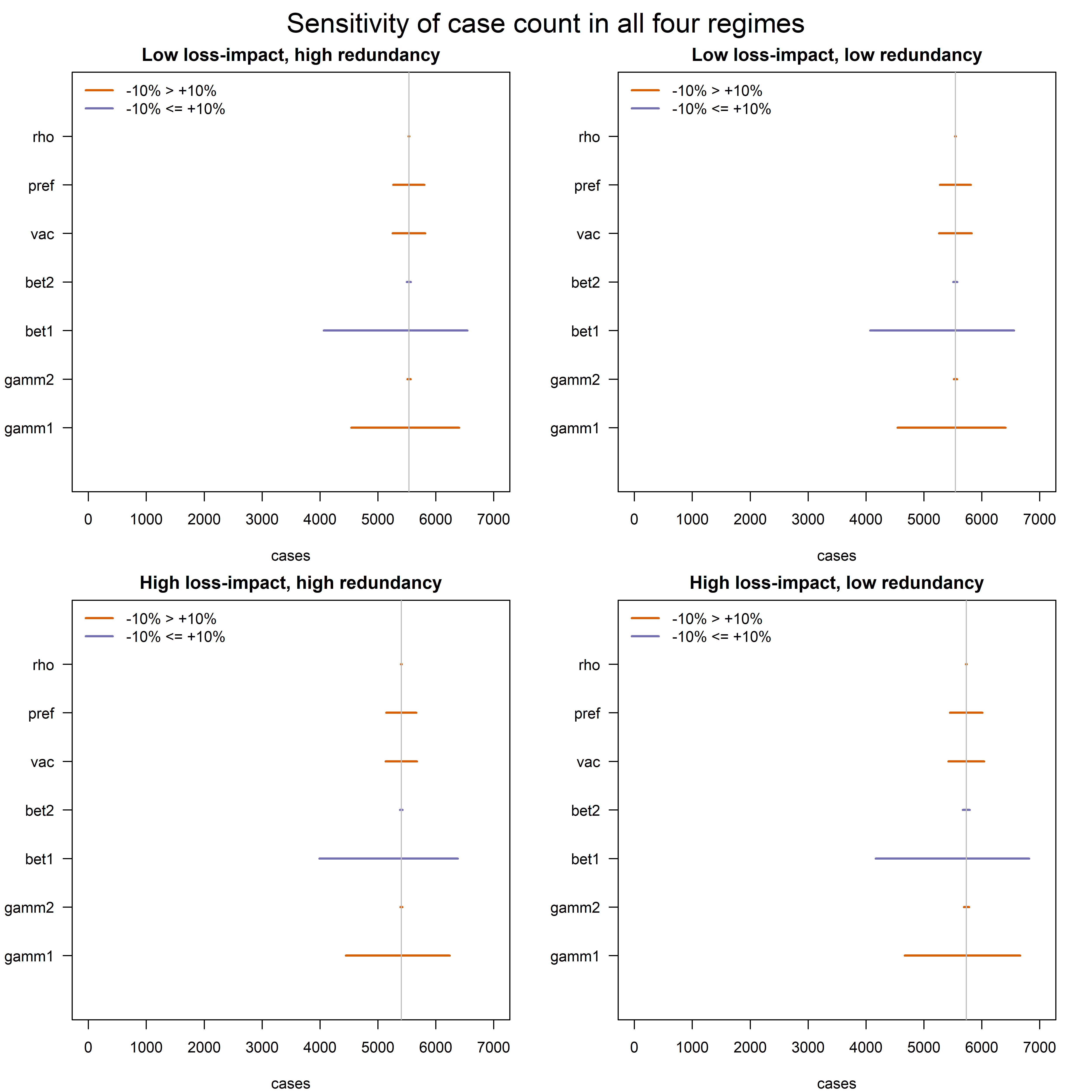


Figure S3: The sensitivity in the final number of cases caused by adjusting all non-Quality of Care parameters up and down by 10%. “High loss impact” refers to a value of k of 4.5, “low loss impact” refers to a value of k of 0.5, “high redundancy” refers to a value of m of 0.9, and “low redundancy refers to a value of m of 0.1. Regardless of the parameterization of quality of care, the number of cases is most sensitive to the transmission rate of the general population (bet1) and the recovery rate of the general population (gamm1). As healthcare workers make up only around 2% of the population, the transmission rate of healthcare workers (bet2) and recovery rate of healthcare workers (gamm2) make relatively little difference to the number of cases. Finally, death rate (rho), vaccine preference for healthcare workers (pre), and vaccination rate (vac) all have relatively little impact. The qualitative pattern is the same regardless of quality of care parameterization.


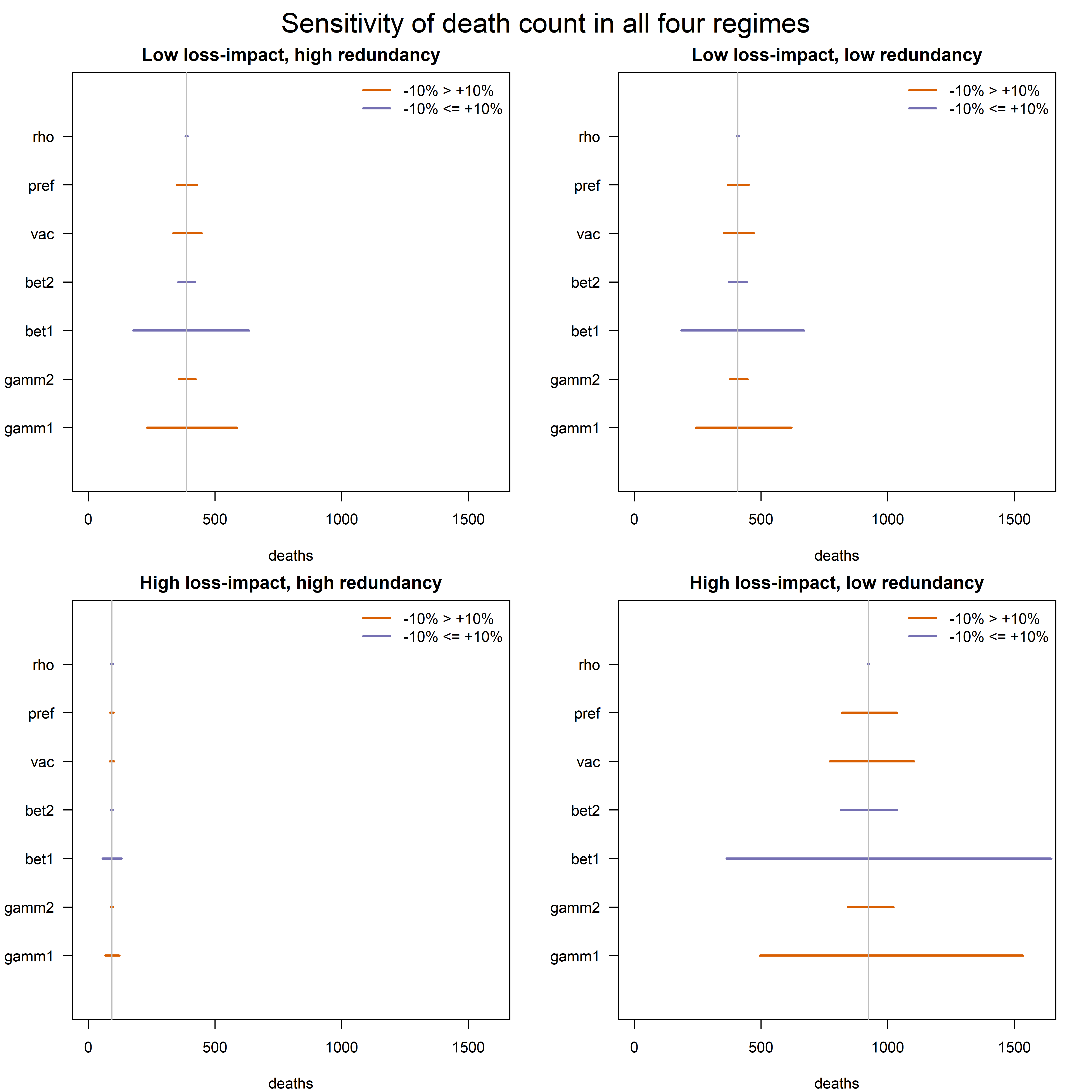
Figure S4: The sensitivity in the final number of deaths caused by adjusting all non-Quality of Care parameters up and down by 10%. “High loss impact” refers to a value of k of 4.5, “low loss impact” refers to a value of k of 0.5, “high redundancy” refers to a value of m of 0.9, and “low redundancy refers to a value of m of 0.1. The number of deaths is also most sensitive to the transmission rate of the general population (bet1) and the recovery rate of the general population (gamm1). The model is most sensitive in high loss-impact, low redundancy situations, which also have the largest death counts. The model is minimally sensitive in high loss-impact, high redundancy situations: if the loss of healthcare workers makes minimal difference to quality of care until a large number are lost, small changes in infection parameters have less impact on the overall mortality.


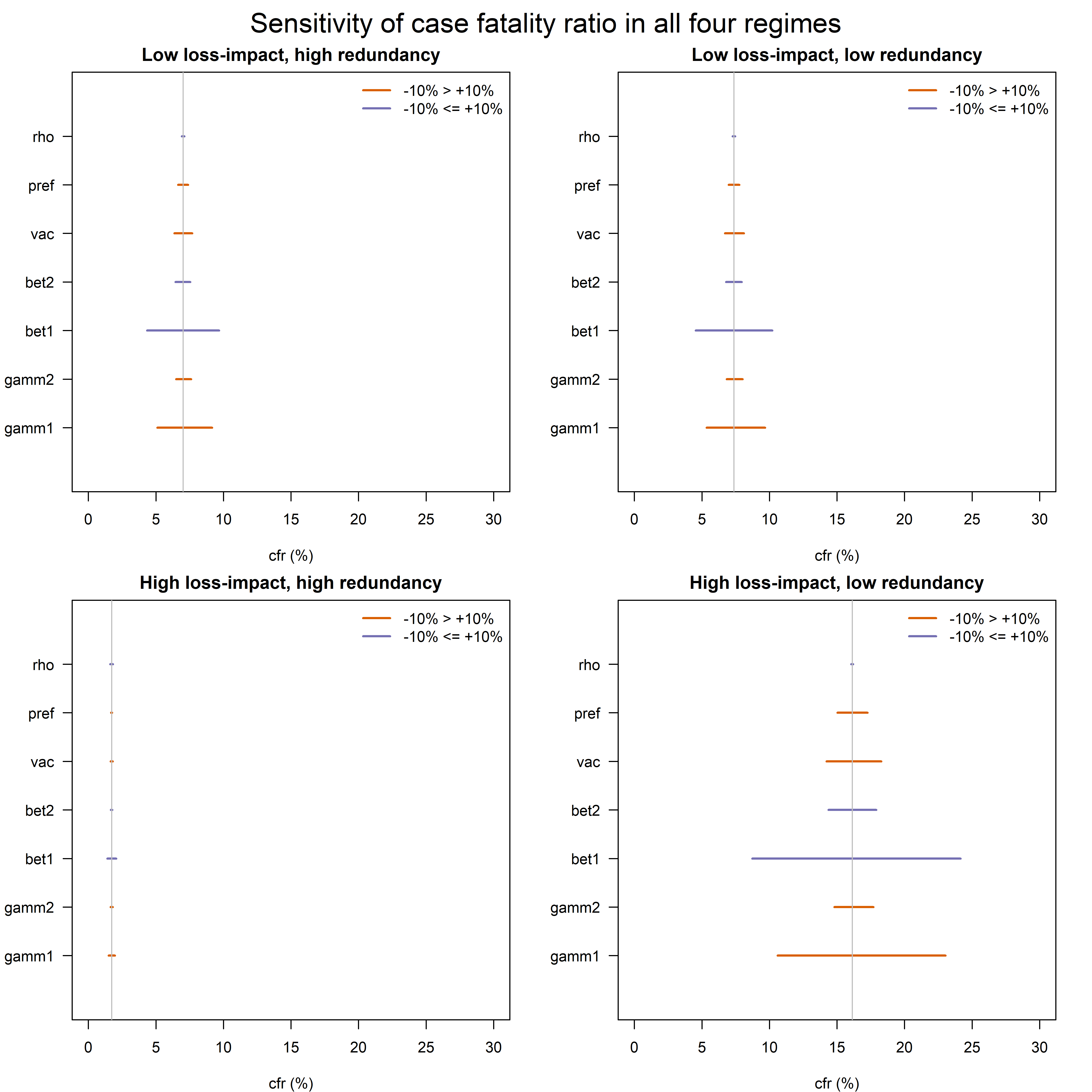


Figure S5: The sensitivity in the case fatality ratio caused by adjusting all non-Quality of Care parameters up and down by 10%. “High loss impact” refers to a value of k of 4.5, “low loss impact” refers to a value of k of 0.5, “high redundancy” refers to a value of m of 0.9, and “low redundancy refers to a value of m of 0.1. The case fatality ratio is also most sensitive to the transmission rate of the general population (bet1) and the recovery rate of the general population (gamm1). The model is most sensitive in high loss-impact, low redundancy situations, which also have the largest case fatality ratios. The model is minimally sensitive in high loss-impact, high redundancy situations: if the loss of healthcare workers makes minimal difference to quality of care until a large number are lost, small changes in infection parameters have less impact on the overall case fatality ratios.
